# *Hymenolepis diminuta* Infection in a Romanian Child from an Urban Area

**DOI:** 10.3390/pathogens11030322

**Published:** 2022-03-07

**Authors:** Felicia Galoș, Mălina Anghel, Andreea Ioan, Mara-Ioana Ieșanu, Cătălin Boboc, Anca Andreea Boboc

**Affiliations:** 1 Department of Pediatrics, Marie Curie Emergency Children’s Hospital, 041451 Bucharest, Romania; felicia.galos@umfcd.ro (F.G.); malina_malina0@yahoo.com (M.A.); berariuandreea@gmail.com (A.I.); catalin.boboc@hotmail.com (C.B.); anca.orzan@umfcd.ro (A.A.B.); 2 Department of Pediatrics, Carol Davila University of Medicine and Pharmacy, 020021 Bucharest, Romania

**Keywords:** *Hymenolepis diminuta*, rodent, seizures, infant, cestode

## Abstract

*Hymenolepis diminuta* is primarily a rodent parasite that is ubiquitously distributed worldwide, but with only a few cases described as human infections. We report a case of *Hymenolepis diminuta* infection in a 15-month-old child, living in an urban setting, with no previous medical history. The patient presented with two episodes of seizures, and complaints of abdominal pain, vomiting, and diarrhea, with no apparent history of rodent contact. Furthermore, the patient’s gastrointestinal symptoms were linked to the emission of suspected tapeworm proglottids in the feces. After excluding other possible etiologies, a diagnosis of *Hymenolepis diminuta* infection was made, based on the examination of characteristic eggs in a concentrated stool specimen. The infant was successfully treated with praziquantel and fully recovered. After two weeks, the stool sample was free of *Hymenolepis diminuta* eggs. The clinical follow-up over the next 3 years was normal. *Hymenolepis diminuta* is rarely found in humans, and, when present, the infection is frequently asymptomatic. Abdominal pain, irritability, itching, eosinophilia, and seizures have also been reported. In this paper, we report, for the first time in the literature, an infection with *Hymenolepis diminuta* in a Romanian infant who had atypical neurological presentation, with full recovery, without subsequent neurological sequelae.

## 1. Introduction

The zoonotic cestode *Hymenolepis diminuta* (*H. diminuta*) is primarily a rat tapeworm, found in the small intestine of rodents, which are the definitive hosts. Humans can enter the tapeworm’s life cycle by accidental consumption of infected insects (intermediate hosts), which contain the helminth’s cysticercoids in their body cavity [1].

*H. diminuta* infection in humans is rare, even in developing countries, typically occurring in isolated cases, such as case reports describing a single affected individual. Surveys of various groups have found infection rates ranging from 0.001 to 5.5%, especially in children [2,3,4,5,6,7,8]. The infection is paucisymptomatic, with pruritus, abdominal pain, and diarrhea as the main manifestations. The diagnosis of *H. diminuta* infection in humans is confirmed by the presence of the characteristic eggs in the stool sample. Cases have been reported all over the world. In Romania, sporadic cases have also been reported [9,10].

Herein, we report an *H. diminuta* infection in an infant from a tertiary center in Romania, presenting with afebrile seizures, with a favorable outcome after receiving antiparasitic drugs. To the best of our knowledge, this is the first documented case of *H. diminuta* infection in a human from Romania, with atypical presentation, such as afebrile seizures.

## 2. Case Presentation

A 15-month-old female infant, living in the urban area of Bucharest, Romania, was referred to the Emergency Department of Marie Curie Emergency Children’s Hospital for two episodes of generalized tonic–clonic seizures, lasting approximately 10 min, and followed by postictal sleep. The length of time between seizures was 30 min. The onset of symptoms was 24 h before the presentation, with vomiting, mild diarrhea, and abdominal pain, due to the emission of suspected tapeworm proglottids in her stool. The patient’s previous medical history was negative, and she had no recent travel history. The subsequent physical examination, complemented by cerebral computed tomography, electroencephalography, and chest X-ray, was normal. No abnormalities were revealed by blood and urine analyses.

The first seizure episode was treated with diazepam. Phenytoin was introduced after the second seizure, with subsequent tapering for 14 days.

Considering the emission of a suspicious worm in the patient’s stool, a parasitological examination of a concentrated stool sample was performed. Direct microscopic examination of the eggs in an unstained moist mount is required for this investigation. To improve the accuracy of diagnosing infections, concentration techniques and multiple tests are needed. The first examination of the stool sample described parasite eggs of unknown identity. The morphological description revealed spherical eggs, 70 μm in diameter, with a striated outer membrane and a thin inner membrane, containing six central hooklets, but no polar filaments. The diagnosis of *H. diminuta* infection was subsequently made after consulting an experienced parasitologist. The identified *H. diminuta* eggs were distinguished from *H. nana* eggs, which have a similar appearance, but are smaller and feature two evident polar thickenings, from which four to eight polar filaments arise. 

Treatment with praziquantel (10 mg/kg/day) was administered for 7 days. After two weeks, the infant was clinically stable and the stool sample was clear of *H. diminuta* eggs. Follow-up over the subsequent 3 years was marked by normal growth, no seizures, and proper neurological development.

## 3. Discussion

### 3.1. Prevalence

The zoonotic cestode *H. diminuta* belongs to the *Hymenolepididae* family [1]. *H. diminuta* infection in humans is uncommon, typically occurring as isolated cases. As a result, only a few hundred cases have been reported, mainly in children [11,12,13,14,15,16,17,18,19,20] and rarely in adults [21,22]. One recent review identified 1561 published records of *H. diminuta* infection in humans, from 80 countries, estimating the total number of human cases worldwide, mostly with no evidence of the existence of infected rodents [1]. Most of the published literature describes isolated case reports. In Romania, only a couple of human infections were documented. According to the latest report, a routine stool examination revealed *H. diminuta* eggs in an asymptomatic 3-year-old girl [9].

In other countries, different situations were reported. For example, in a 5-year-old girl infected with *H. diminuta*, with occasional abdominal pain and anal pruritus, the authors reported cyanosis, loss of consciousness, stiffness of the limbs, followed by drowsiness and hypotonia induced by crying, according to their description [14]. In 1989, a child from Jamaica became the first documented case of *H. diminuta* in a human in Jamaica, West Indies [23]. *H. diminuta* eggs were also discovered in a 12-year-old girl residing in a small village in rural India, an area extensively infested with rats and cockroaches [11]. In addition, in an urban area of Rome, a 2-year-old boy was infected with *H. diminuta*. In this case, however, the investigators found no evidence of rodent or other suspected sources of infection [13], similar to our situation. All these examples emphasize the rarity of *H. diminuta* infection and its worldwide distribution.

### 3.2. Life Cycle

First, *H. diminuta* eggs are passed from the infected definitive host’s feces (e.g., rodents, man). Intermediate hosts, such as arthropods, consume the mature eggs, and oncospheres are released, piercing the host’s intestinal wall. These develop into cysticercoid larvae, which survive as the arthropod develops into adulthood. The mammalian host contracts *H. diminuta* infection after ingesting a cysticercoid larvae-carrying intermediate host. Humans can become accidentally infected by eating insects from precooked cereals or other foods, as well as directly from the environment (e.g., oral exploration of the environment by children). The contaminated arthropod’s tissue is degraded after ingestion, releasing cysticercoid larvae into the stomach and small intestine. The scolexes are everted shortly after the cysticercoid larvae are expelled.

The parasite attaches to the small intestinal wall using the four suckers on the scolex. The worms mature in 20 days, and adult parasites can grow up to 30 cm in length. Tapeworms are hermaphrodites, and each proglottid carries a set of female and male reproductive organs. Gravid proglottids release eggs into the small intestine, which disintegrate after breaking off from the adult parasites. Finally, the eggs are released into the environment through the feces of the mammalian host [24].

In humans, *H. diminuta* infection spreads by the consumption of infected intermediate hosts. *H. diminuta* cysticercoids have been found in more than 30 insect species, including moths, grain beetles, and fleas, as well as other arthropods, such as millipedes [25]. However, due to variances in their distribution, abundance, capability to be infected in multiple phases of their life cycle, and adaptation to survive in anthropogenic ecosystems, it is rather unclear if these insects play a substantial role in the transmission of *H. diminuta* to humans.

### 3.3. Symptoms, Treatment, and Outcome

Most human infections with *H. diminuta* lead to gastrointestinal symptoms, such as abdominal pain and diarrhea, but also fever [1,12,17,18,19,20]. Extraintestinal symptoms, including pruritus, irritability, and arthromyalgia, have also been associated with *H. diminuta* infection in some cases [1,11,13,14]. A fatal outcome has been reported in an adult patient. However, the infection coexisted with an underlying intra-abdominal malignancy, which made the fatal outcome more likely [21].

*H. diminuta* infection may also cause eosinophilia, due to mucosal damage to the intestinal villi [1,14,18], but reports on this are inconsistent [1,19].

In terms of therapy, there is no clear evidence of its effectiveness, due to a lack of controlled studies and evidence, suggesting that many infected and untreated patients become asymptomatic over time. Nonetheless, for the treatment of *H. diminuta* infection, praziquantel seems to be the drug of choice [11,12,15,16,17,18,26], even though its safety profile in the pediatric population is not well established. Niclosamide is a treatment alternative given to children especially, but there is low availability of this drug in many countries [13,14,21,27,28].

### 3.4. Hymenolepis Diminuta and Nana Difference

Hymenolepiasis is, in fact, caused by two cestodes species, *H. diminuta*, the rat tapeworm, and *H. nana*, the dwarf tapeworm. In humans, infection with *H. nana* is much more common than infection with *H. diminuta. H. nana* is probably the tapeworm encountered most in humans, since it can be directly propagated from one person to another, having no need for an intermediate host [29]. Direct human-to-human transmission appears to be the most prevalent route of infection, especially in environments where poor hygiene and inadequate sanitation are present [30,31]. These cestodes have almost identical clinical presentations and alike treatment strategies, with praziquantel being the treatment of choice.

**Figure 1:**
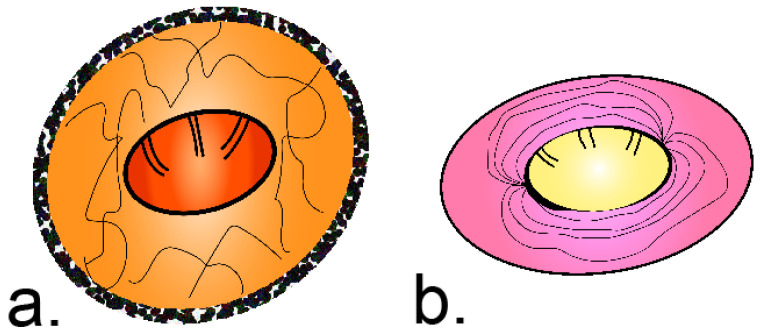
The morphology difference between *H. diminuta* and *H. nana* eggs. (**a**) Egg of *H. diminuta*—round or slightly oval, with a size of approx. 70 µm × 80 µm, with a thick striated outer membrane and a thin inner membrane; the oncosphere has 6 central hooks (hexacanth). (**b**) Egg of *H. nana*—oval or subspherical, with a smaller size of approx. 40 µm × 30 µm; the inner membrane has two poles, from which 4 to 8 polar filaments spread out between the membranes; the oncosphere also has 6 central hooks (hexacanth).

The diagnosis of hymenolepiasis is indicated by the presence of cestode eggs in the stool sample, under a microscope. *H. nana* eggs are different from those of *H. diminuta,* in terms of size, polar thickenings, and filaments (Figure 1) [32].

### 3.5. Hymenolepis Diminuta and Immunity

An interesting feature of *H. diminuta* infection is that it can act as a promising therapeutic candidate to alleviate inflammatory diseases, which have proven to be resistant to pharmacological interventions. Infection occurs through ingestion, and the worm does not migrate through the host; rather, it remains in the small intestine. Bearing no teeth or hooks, it causes no obvious abrasive damage to the host. It is not auto-infective, as its life cycle requires an intermediate arthropod host, so there is no direct person-to-person spread [33].

This theory is supported by the fact that helminths have evolved to manipulate their hosts’ immune systems [34]. Indeed, animal studies suggest that helminth parasite infection lowers the severity of inflammatory diseases [35,36,37,38,39]. In chronic worm infections, allergic symptoms are common, owing to the persistent state of immunological activation, defined by dominating Th2-type cytokines, including interleukin-10, which has an anti-inflammatory effect, and IgE-mediated responses [40]. Infections with parasitic helminths induce Th2-dominated immunity, while pro-inflammatory Th1 cytokine production is maintained at low levels [40].

### 3.6. Case Particularities

In the present case, the patient came from an urban area, and the house and its surroundings, as well as the areas frequently visited by the infant, had no evidence of rodents or other possible sources of infection. This may indicate the possibility of another indirect mechanism of transmission, such as food grains from a rat-infested mill or drinking water contaminated with intermediate hosts, adding difficulty to the diagnosis.

The particularity of this case originates from the rarity of *H. diminuta* infection in humans in Romania, along with the particular presentation. Furthermore, because this infection is more often encountered in tropical and subtropical areas, and Romania has a temperate climate, this occurrence is all the more surprising.

Moreover, the patient had an atypical presentation, with afebrile seizures. Although the first differential diagnosis targeted the causes of seizures during infancy, the results came back negative. The relationship between seizures and *H. diminuta* infection was difficult to establish because this symptom was unusual. The latest worldwide review of *H. diminuta* infections did not include seizures as a clinical sign [1]. A single case report was found in the literature describing febrile seizures in a child with *H. diminuta* infection; however, the patient had a positive medical history for febrile seizures [41].

Regarding laboratory analysis, *H. diminuta* infection can cause eosinophilia, but in our patient, this finding was not detected.

Praziquantel is the treatment of choice for *H. diminuta* infection. To ensure parasite eradication, our patient was prescribed praziquantel (10 mg/kg/day) for 7 days, with a good safety profile. The following parasitological examination of the stool revealed no eggs, suggesting that the treatment had high efficacy. However, currently, therapeutic handbooks do not exactly cover the treatment of *H. diminuta*, possibly due to its low prevalence. Therefore, given the lack of information regarding praziquantel treatment, every case of *H. diminuta* infection should be recorded, particularly data on treatment procedures, and parasitological and clinical outcomes.

## 4. Conclusions

We report, for the first time, an *H. diminuta* infection in a Romanian infant with atypical manifestations, such as generalized seizures and non-specific gastrointestinal symptoms, who made a full recovery following praziquantel treatment. This case report highlights that a precise parasitological diagnosis requires proper district laboratories and qualified personnel.

To increase our understanding regarding the epidemiology, mode of transmission, and therapeutic protocol, and to improve the management of this rare infection, we recommend that every *H. diminuta* infection in a human should be reported and investigated.
